# Tensile and Bending Strength of Birch and Beech Lamellas Finger Jointed with Conventional and Newly Developed Finger-Joint Profiles

**DOI:** 10.3390/ma17205063

**Published:** 2024-10-17

**Authors:** Hannes Stolze, Holger Militz

**Affiliations:** Wood Biology and Wood Products, Faculty of Forest Sciences and Forest Ecology, University of Goettingen, Buesgenweg 4, 37077 Goettingen, Germany; hannes.stolze@uni-goettingen.de

**Keywords:** adhesives, beech, bending strength, birch, finger-joint profile, tensile strength

## Abstract

In this study, the tensile and bending strength of birch and beech lamellas finger jointed with conventional (*Standard*) and newly developed finger-joint profiles (*New*) are presented. Polyurethane (PUR), Melamine-Urea-Formaldehyde (MUF) and Phenol-Resorcinol-Formaldehyde (PRF) adhesive systems were used to bond the finger joints. The objective of the *New* profiles was to reduce the stress concentrations within the finger joint by cutting the cross-grooved fingers perpendicular to the main orientation of the finger-joint profile. In the first trials of the development, larger cross-grooved fingers were cut with the aim to improve the stress distribution and to reinforce the finger joint by filling gaps in the finger joint with adhesive. As the study progressed, initial optimisations of the *New* profile were made. Smaller cross-grooved fingers were cut as it was assumed that they are beneficial for the manufacturing and integrity of the *New* profile. In combination with the MUF adhesive system, the *New* profile achieved the highest increase in the bending and tensile strengths compared to the *Standard* profile. In addition to the increased strength, other advantages such as reduced cracking in the finger joint were observed when using the *New* profile. The high strength and stiffness of hardwoods or other high-performance materials used in timber construction can probably be better exploited in combination with the *New* profile. Further tests will be carried out by considering different configurations of the *New* profile and different materials.

## 1. Introduction

In ref. [1], several approvals for hardwood engineered wood products (EWPs) were granted by building authorities [2,3], which led to hardwood becoming increasingly usable for engineers, architects, etc., in the construction industry. To date, only a few hardwood EWPs have entered the market, and, overall, they play a minor role in timber construction. The high strength and stiff wood species beech (*Fagus sylvatica*, L.), which is widespread in central Europe, has been comprehensively researched for use in EWPs. The high availability, high mechanical potential and, so far, high proportion of energy use favour the increased use of EWPs made from beech. Recently the wood species birch (*Betula pendula*, Roth and *Betula pubescens*, Ehrhart), has gained interest in Germany as it has a high potential as a structural material due to its high strength and stiffness. Birch is mainly distributed in Scandinavian countries, Russia and Baltic countries [4,5]. In Germany, birch received little attention in forest management in the past. Today, it is being established in a natural way in forest stands that have suffered from climate change, and large-sized birch logs can generally be produced within relatively short periods.

**Figure 1:**
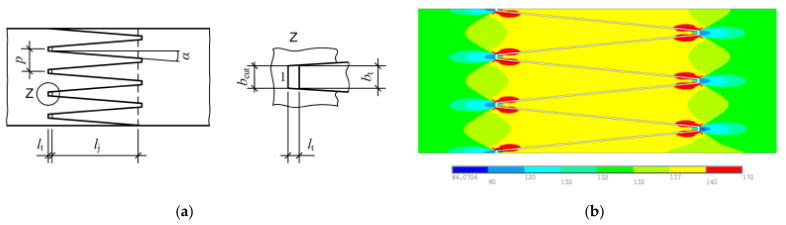
Typical profile of a structural finger joint according to EN 15497 [11]. Finger length (l_j_), finger pitch (p), finger angle (α), tip gap (l_t_), tip width (b_cut_) and finger base (Z) (**a**). The distribution of the longitudinal stresses in a finger joint (stresses increase from blue to red) [12] (**b**).

Finger jointing, which was developed in the 1930s, is one of the main steps in EWP production. Finger joints are important for the performance and economic efficiency of EWPs. They enable product dimensions far beyond the dimensions of raw timber [6]. In the past, finger jointing was mainly standardised using spruce (*Picea abies*, L.), which is the dominant wood species in timber construction [7,8]. The adhesive systems and finger-joint geometry used (referred to as a profile in this study) have a major influence on the properties of a finger joint [9]. The use of new technologies in wood processing, such as scanner technology, makes it possible to determine and eliminate defects in the wood more effectively. In addition, as already mentioned, large quantities of hardwoods are available whose strengths and stiffness can far exceed those of spruce. However, up to now, hardwoods have rarely been used for load-bearing structures. In contrast to softwood, the finger joint in hardwoods is often the decisive factor for the strength of an EWP [10]. In a load case, conventionally used structural finger joints have stress concentrations, especially in the base of the finger joint and at the tip of the fingers (Figure 1), which often lead to the failure of the joint.

In accordance with the standards EN 15497 [11] and EN 14080 [13], structural finger joints require a tip gap (l_t_) in the finger joint. The tip gap serves to form a thin bondline and to achieve a self-locking of the finger joint. However, it also leads to a weakening of the timber cross-section in the base (Z) of the finger joint [9]. Furthermore, the joining process is associated with a prestressing, which is noticeable after a pressing of the joint or in later use in the form of cracks [14,15,16]. The cracks usually originate from the base of the finger joint, and they are often located at the edges of the finger-jointed lamellas. Studies concerning the stress distribution in conventionally used finger joints were carried out by [17], and weak points were identified whose influence increased with higher loads. Other studies [9,12,18,19,20,21,22] have also carried out investigations into the stress behaviour of finger joints. These authors pointed out that the low strengths of finger joints often result from stress concentrations in the base of the finger joint that overlap with the adjacent fingers.

**Figure 2:**
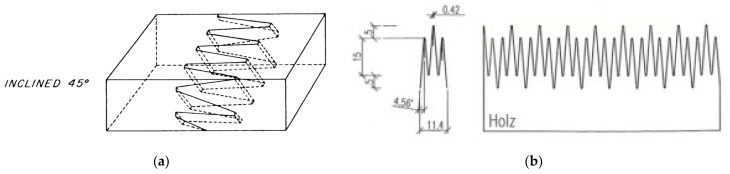
Schematic of modified finger-joint profiles, inclination of the finger-joint profile according to [25] (**a**) and shifted fingers according to [18,23] (**b**).

The finger joint has been continuously developed, and a variety of finger-joint profiles have been designed for different applications. A changing mix of resources and the development of new EWPs foster the need for further adaptation and optimisation in finger jointing. The following authors have shown that the strengths of the finger joint can be increased by optimising the finger-joint profile [18,22,23,24,25,26]. The optimised profiles in [24] led to an increase in the bending strength of the finger-jointed beech lamellas when a larger finger length (l_j_) and a smaller finger pitch (p) were used. Ref. [18] optimised the finger-joint profile by shifting the fingers to avoid stress overlaps. The distance between the stress concentrations was increased, and the bonding surface was kept constant. The same principle as in [18] was used for the modified profile in [23]. Shifted fingers were manufactured with the aim to eliminate the stress bridging and to achieve a high bonding surface (Figure 2). The modified profile in [23] led to an increase in the bending strength but not in the tensile strength. In addition, ref. [24] carried out tests with an inclined finger-joint profile, which led to a slight increase in bending strength in some cases.

From an economic point of view, the manufacturability and yield of a finger-joint profile must be considered in addition to the overall performance of the finger joint.

In recent years, a small number of adhesive systems have been approved for the structural bonding of hardwoods [10]. Hardwoods and new materials bring with them new requirements. Further optimisation of the conventional finger-joint technology is needed [23]. The aim of this study was to develop adjusted finger-joint profiles and to determine the tensile and bending strengths of the finger-jointed lamellas. High-strength hardwood lamellas and structural adhesives were used for the tests.

## 2. Materials and Methods

**Figure 3:**
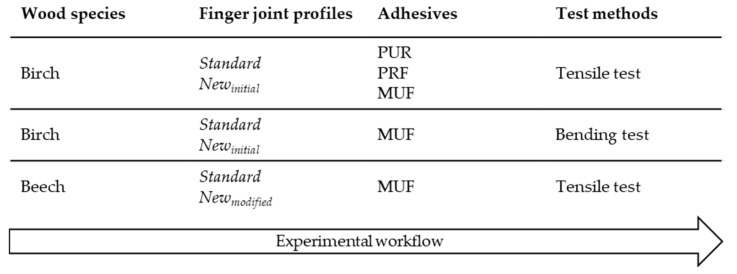
The experimental workflow of this study.

Figure 3 illustrates the experimental workflow of this study, and further explanations are made in the following sections.

In this study, birch wood (*Betula pendula*, Roth) originating from Aizkraukle, Latvia, and beech wood originating from Göttingen, Germany, were used. The birch planed lamellas (2000 × 150 × 35 mm^3^, length × width × thickness, predominantly tangential grain) were produced in the sawmill of SIA Krauss LTD, Madonas novads, Latvia, and were kiln-dried. The planed beech lamellas (2000 × 100 × 25 mm^3^, mix of grain orientations) were produced in the Wood Biology and Wood Products Department in Göttingen, Germany, and air-dried in the technical lab for several months. Overall, the lamellas were of high quality and almost without knots. The beech lamellas showed more pronounced grain deviations than the birch lamellas. Commercial Melamine-Urea-Formaldehyde (MUF), Phenol-Resorcinol-Formaldehyde (PRF) and 1-component Polyurethane (PUR) adhesive systems were used to bond the finger joints. The adhesives were processed according to the technical data sheets of the adhesive manufacturers (Table 1). Higher application quantities were used in some cases for the newly developed profiles (*New*). This is further indicated in the corresponding sections.

### 2.1. Determination of the Lamella Properties and Group Formation

The properties of the lamellas that were used in tensile tests were determined before finger jointing. The moisture content (MC) was measured at three points of each lamella using a capacitive wood moisture meter FMD 6 (Brookhuis, Enschede, Netherlands) and were averaged. The density (ρ) was determined by dividing the mass by the volume of the lamellas. The dynamic modulus of elasticity before finger jointing (E_0_) was determined on both ends of the lamellas by means of a Timber Grader (MTG, CE marking according to EN 14081 [27], Brookhuis, Enschede, Netherlands) and were averaged. Groups with similar mean E_0_ and number of lamellas were formed. The groups were differentiated according to which adhesive (PUR, MUF and PRF) and finger-joint profile—conventional (*Standard*, *S*) or newly developed (*New*, *N*)—they were to be finger jointed. The groups were referred to as unjointed reference (Control), PUR *Standard* (PUR*S*), PUR *New* (PUR*N*), MUF*S*, MUF*N*, PRF*S* and PRF*N*. Before finger jointing, the lamellas were cut in the middle, and both halves were marked to guarantee that the lamella halves can be joined together as they were in original state.

### 2.2. Production of the Finger-Joint Profiles

An Ultra TT finger-jointing line from Weinig Grecon GmbH & Co. KG (Alfeld/Leine, Germany) was used to produce the finger-jointed lamellas. The finger-joint profiles were cut by means of a finger-joint cutter (Leuco AG, Horb a. N., Germany), which is recommended for structural finger joints according to EN 14080 [13]. The finger pitch (p) of the cutter was 3.8 mm, and the finger length (l_j_) was variable between 15–16.5 mm. The finger-jointing line was equipped with an additional cutting device that could shorten the finger length beyond the minimum finger length of the finger-joint cutter. All finger joints of this study were cut with a feed rate of 17 m·min^−1^, the adhesives were manually applied on both lamella halves and the joints were pressed for 5 s with 11 N mm^−2^ pressure for the birch lamellas and 15 N mm^−2^ for the beech lamellas. The pressing pressure of the beech lamellas was higher due to the higher density of the beech compared to birch.

**Figure 4:**
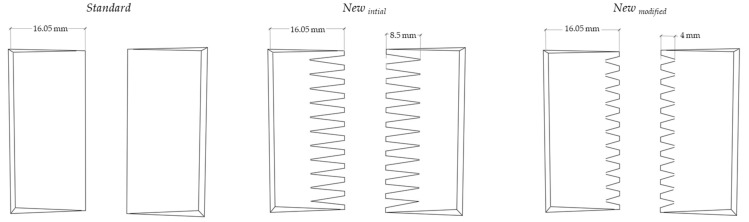
Sketches of the produced finger shapes and their finger length.

The three finger shapes of the finger-joint profiles *Standard, New_initial_* and *New_modified_* shown in Figure 4 were produced and will be explained in more detail in the following sections.

#### 2.2.1. Conventional Finger-Joint Profile

The conventional finger-joint profile, referred to as *Standard* (*S*) profile, was cut with the vertical finger joints showing the fingers on the wide face of the lamellas. The vertical orientation of the finger joint is common for structural finger joints in glued laminated timber. The vertical finger length was set to 16.05 mm (±0.05 mm) for all lamellas in this study and was the main orientation of the finger-joint profiles. The profile of the second half of the lamella was cut with a height shift of 1.9 mm (finger pitch p/2) to enable the lamella halves to be joined without offsets. 

#### 2.2.2. Newly Developed Finger-Joint Profiles

In Part 1 of the study, the aim of the newly developed finger-joint profile, referred to as *New_initial_* profile, was to improve the stress distribution of the finger joint. In addition, it was intended to reinforce the finger joint due to the adhesive filled gaps that laterally cross the *New_initial_* profile.

**Figure 5:**
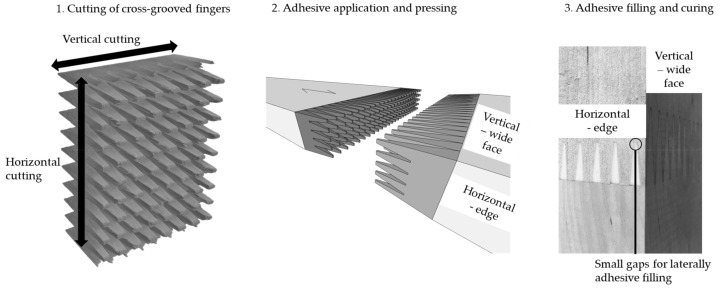
Manufacturing of the newly developed *New_initial_* profile.

Figure 5 shows a schematic of the manufacturing of the *New_initial_* profile.

The *New_initial_* profile combined horizontal and vertical cuttings that were oriented crosswise at the end face of the lamellas. The horizontal cutting notched the vertically aligned fingers at regular intervals. The fingers of the *New_initial_* profile are referred to as cross-grooved fingers. Stresses were supposed to be reduced compared to continuous fingers due to a lower notch effect of the cross-grooved fingers. In this study, at first, horizontally oriented fingers were cut showing the fingers on the edge of the lamellas. They were cut equally for both lamella halves without a height shift of the cutter. Then, the lamellas were cut vertically, as previously described for the *Standard* profile, whereby the horizontally oriented fingers were shortened with the additional cutting device of the finger-jointing line. The finger length was 8.5 mm (±0.05 mm) horizontally and 16.05 mm (±0.05 mm) vertically. Horizontal cutting without a height shift of the cutter was used to produce small gaps, which were at the same height in the finger joint. After a pressing of the joint, the gaps had a length of about 0.5–1.0 mm. The intention was that the adhesive would laterally spread through the gaps during pressing and would fill them. Table 2 shows the series of birch lamellas finger jointed with the *Standard* and *New_initial_* profile that were tested in tensile tests.

The adhesive application quantity for birch lamellas finger jointed with the *Standard* profile was 160 g m^−2^ for PUR (PUR*S*160) and 350 g m^−2^ for MUF and PRF (MUF*S*350 and PRF*S*350). According to EN 14080 [13], adhesive must wet the fingers and leak out of the finger joint during pressing. As the *New_inital_* profile contained more gaps, and to guarantee an adhesive leakage, twice the amount of adhesive was applied for the series of the *New_initial_* profile PUR*Ni*320, MUF*Ni*700 and PRF*Ni*700 compared to the *Standard*. In total, 94 birch lamellas were cut in the middle and were finger jointed as in their original state. Then, 16 birch lamellas were tested as an unjointed reference (Control). After finger jointing, the dynamic MOE (E_0,fj_) was determined again on 65 finger-jointed lamellas.

In Part 2 of the study, the bending strength and global modulus of elasticity (MOE) in the bending of birch finger-jointed specimens (655 × 31 × 31 mm^3^) were tested (Table 3).

The specimens were produced after the completion of the tensile strength tests of Part 1. Overall, the birch finger-jointed lamellas bonded with MUF achieved the highest tensile strengths. Hence, the MUF was selected to produce the bending test specimens. The specimens of the series were manufactured from the same birch lamellas. The production of the *Standard* and *New_intial_* profiles was as in Part 1 except for the specimen dimensions and adhesive application quantity. The bending test specimens with a *New_initial_* profile were manufactured with 350 g m^−2^ (MUF*Ni*350) and 700 g m^−2^ (MUF*Ni*700), and the *Standard* profile was manufactured with 350 g m^−2^ (MUF*S*350). At least 30 bending test specimens were tested in each series. The density (ρ) and MC was determined after the bending test by means of 31 × 31 × 31 mm^3^ specimens in accordance with DIN 52182 [28] and EN 13183 [29].

In Part 3 of the study, the first adaptations of the *New_initial_* profile were made and finger-jointed beech lamellas were tested in tensile tests (Table 4). Smaller cross-grooved fingers were cut as it was assumed that they are beneficial for the manufacturing and integrity of the *New* profile. The adjusted profile was referred to as the *New_modified_* profile. The finger length was 4 mm (±0.05 mm) horizontally and 16.05 mm (±0.05 mm) vertically. MUF with an application quantity of 350 g m^−2^ was used for finger jointing the beech lamellas.

### 2.3. Determination of the Tensile and Bending Strength

The strength determination was carried out after adhesive curing. The tensile strength of the finger-jointed lamellas parallel to the grain (f_t,0_) was determined according to EN 408 [30] using a high-capacity tensile testing machine (GEZU 600, load cell 600 kN, funded by the German Research Foundation—INST 186/1380-1 FUGG) from the manufacturer Zum Wald (Erlenbach, Switzerland). The finger-jointed lamellas were clamped on each side with a clamping area of 150 × 600 mm^2^ and a clamping pressure of 3.5 N mm^−2^ (Figure 5). The free test span between the clamps was 700 mm, and the finger joint was centred. The test speed was set such that a specific tensile strength—45 N mm^−2^ for birch lamellas and 50 N mm^−2^ for beech lamellas—was reached after 300 s. The test was started when a preload of 5 N mm^−2^ was reached. The location of the failure in the lamellas was documented. Only lamellas showing a failure in the finger joint were used for the evaluation of the finger-joint profiles.

The tensile strength *f*_*t*,0_ was calculated according to Equation (1):

(1) $$f_{t,0}=\frac{F_{\max}}{A},$$

where

*f_t,_*_0_ = Tensile strength parallel to the grain [N mm^−2^];
*F_max_* = Maximum load [N];
*A* = Cross-sectional area [mm^2^].

**Figure 6:**
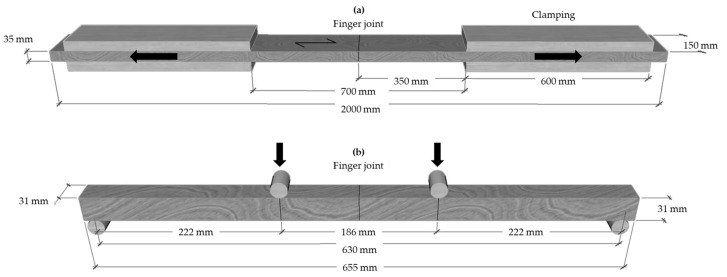
The test setup of the tensile (**a**) and bending strength tests (**b**) with the dimensions of the birch lamella specimens.

The bending strength of the finger-jointed specimens (f_m_) was determined according to EN 408 [29] by means of a 4-point bending test (Figure 6). A universal testing machine (load cell 100 kN, software testXpert III) from the manufacturer Zwick Roell (Ulm, Germany) with a test speed of 5 mm min^−1^ was used. The two upper loading rollers had a span of 222 mm, and the two lower supporting rollers had a span of 630 mm. The global bending modulus of the elasticity of the finger-jointed specimens (*E*_0_*_,fj_*) was determined in a load range between 500–2000 N using a contactless Video extensometer from Zwick Roell. The bending strength *f_m_* and the global bending MOE *E*_0_*_,fj_* were calculated according to Equations (2) and (3):

(2) $$f_{m}=\frac{3Fa}{{bh}^{2}},$$

(3) $$E_{0,fj}=\frac{3al^{2}-4a^{3}\;}{2{bh}^{3}\left[2\frac{w_{2}-w_{1}}{F_{2}-F_{1}}-\frac{6a}{5Gbh}\right]}$$

where

*f_m_ =* Bending strength [N mm^−2^];
*E*_0_*_,fj_ =* Global bending modulus of elasticity of finger-jointed specimens [N mm^−2^];
*F =* Load [N];
*a =* Distance between a load point and the next support in bending test [mm];
*b =* Width of the specimen in bending test or smaller cross-sectional dimension [mm];
*h =* Height of the specimen at bending test or the larger cross-sectional dimension [mm];
*l =* Span in bending test [mm];
*w*_2_ − *w*_1_ *=* Deformation increase corresponding to *F*_2_ − *F*_1_, [mm];
*F*_2_ − *F*_1_ *=* Load increase in the linear range of the load–deformation curve [N];
*G =* Shear modulus [N mm^−2^].

Statistical data analysis was carried out using Microsoft Excel (Excel 2016). Box plot diagrams and simple linear regressions were created. Microscopic images of exemplary *New_initial_* profiles were taken using a VHX-7000 digital microscope (Keyence, Osaka, Japan). The spread of the adhesives within the *New_initial_* profile was visually inspected by means of the images. In addition, the manufacturability of the *New* profiles was assessed based on the cutting behaviour, self-locking, adhesive leakage and cracking. The cracks in the finger joints that were ≥2 mm in length were counted for the beech lamellas that were finger-jointed with the *New_modified_* profile in Part 3 of this study.

## 3. Results and Discussion

**Figure 7:**
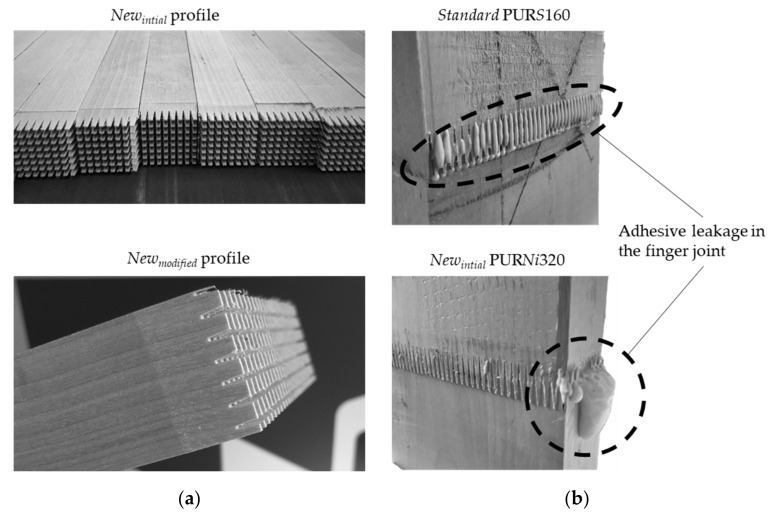
*New* profiles after cutting (**a**), and the adhesive leakage in the finger joint with the *Standard* and *New_intial_* profiles (**b**).

Figure 7 shows the *New* profile after cutting and the *New_initial_* profile after pressing compared to the *Standard* profile. PUR was applied with 160 g m^−2^ on the *Standard* profile and 320 g m^−2^ on the *New_intial_* profile.

After visual inspection, minor tear outs were observed in the *New_initial_* profile when cutting larger cross-grooved fingers. The structural integrity of the *New_modified_* profile was higher as no more tears outs were detected. It was expected that the integrity of the fingers would be mainly affected by the wood species, quality, cutter sharpness and cutting speed. In general, knot and grain deviations in the surrounding of the finger joint can lead to pronounced tear outs.

The *New_intial_* profile mainly showed adhesive leakage on the edges of the lamellas. The gaps that crossed the profile served to laterally spread the adhesive. A large amount of the applied adhesive was pressed out of the *New_initial_* profile, indicating an overdosage, especially in Part 1 of this study. The adhesive leakage of the *Standard* profile was mainly visible on the wide face of the lamellas. The *New_modified_* profile hardly showed any adhesive leakage, neither on the edge nor on the wide face of the lamellas. On the one hand, the adhesive leakage after pressing ensured that sufficient adhesive had been applied and that the adhesive was processable. On the other hand, increased adhesive leakage should be minimized due to economic reasons. Adhesive on the wood surface must be removed in further processing steps. With today’s adhesive application systems, the adhesive application is monitored inline without contact and the application quantity is precisely dosed. The *New_modfied_* profile can be advantageous in terms of production due to the adhesive-free wood surface after pressing. However, there is also a risk that not all gaps are completely filled with adhesive and need further control.

**Figure 8:**
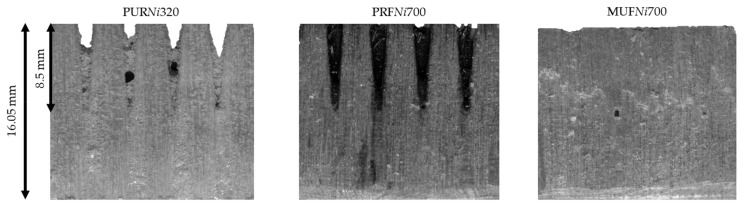
Adhesive filling within the *New_initial_* profile and sample images of the test specimens, which were considered representative of the adhesives, after tensile testing.

The adhesive filling of the cross-grooved fingers in the *New_initial_* profile is shown as an exemplar in Figure 8.

The application quantities used in the studies were different for the different adhesives (PUR 320 g m^−2^, MUF 700 g m^−2^ and PRF 700 g m^−2^). PRF*Ni*700 and MUF*Ni*700 filled a high proportion of the cross-grooved fingers. The PUR*Ni*320 filled the profile incompletely, and numerous larger voids were visible within the cross-grooved fingers. The viscosities of PRF and MUF were lower compared to PUR (Table 1). Most likely, the higher flowability and application quantity of PRF and MUF more completely contributed to filling the gaps during pressing. At the same time, a large proportion of the adhesive was pressed out from the edges of the *New_initial_* profile.

Overall, fewer cracks were observed in the finger joint by means of the *New* profiles compared to the *Standard* profile. The beech lamellas finger jointed with the *Standard* profile showed, on average, 1.4 cracks at a ≥2 mm length in the finger joint, and the lamellas finger jointed with the *New_modified_* profile were, on average, 0.4 cracks at a ≥2 mm length. In further studies, the extent and location of cracks should be considered and quantified. It is suspected that the cross-grooved fingers of the *New* profiles led to a more even stress distribution and fewer stress concentrations in the finger joint. This assumption needs to be further verified by means of stress analyses as it was carried out in [21,31,32]. The self-locking of the finger joints was apparently not influenced by the cutting of the cross-grooved fingers. However, more in-depth tests should also be carried out on this.

In the tensile strength tests, homogeneous birch lamella groups were compared (Table 5). The MC, density and dynamic MOE before finger jointing (E_0_) were similar between the series. The dynamic MOE that was determined after finger jointing (E_0,fj_) showed an increase in all series. In [33,34], it was found that dynamic MOEs exhibit a nearly perfect linear relationship with static (global and local) MOE. A high linear relation between tensile strength (f_t,0_) and E_0_ was found for MUF*Ni*700 (R^2^ = 0.72), and a moderate linear relation was found for the Control series (R^2^ = 0.56). The other test series showed no or a low linear relation between f_t,0_ and E_0_. Most of the finger-jointed lamellas failed in their finger joint.

**Figure 9:**
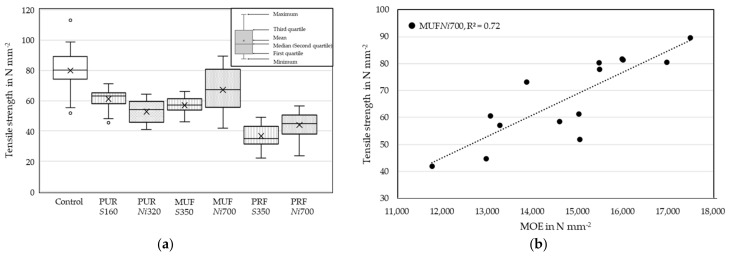
The tensile strength of the finger-jointed birch and Control lamellas (**a**). The relation between the MOE before finger jointing and the tensile strength of the MUF*Ni*700 lamellas (**b**).

Figure 9 shows the f_t,0_ of the finger-jointed birch lamellas and the Control series. In addition, it shows the linear relation between the E_0_ and f_t,0_ of the MUF*Ni*700 finger-jointed lamellas.

Overall, the Control achieved the highest mean f_t,0_ (79.9 N mm^−2^). The test duration of 300 (±120) seconds was exceeded by the Control lamellas in most cases and was often near the upper limit for the finger-jointed lamellas. MUF- and PUR-bonded finger joints achieved significantly higher f_t,0_ compared to PRF-bonded finger joints. A long-lasting formaldehyde smell was noted for the PRF finger-jointed lamellas, indicating an incomplete curing reaction. A too low hardener content or too low temperature for the curing reaction of the PRF could explain the difference but could not be clarified retrospectively. In the study of [35], the PRF bond strengths in birch finger joints were similar to the MUF bonds. Therefore, similar strength properties of the adhesives were expected in this study. Lamellas finger jointed with MUF and PRF achieved a higher mean f_t,0_ with the *New_initia_*_l_ profile compared to the *Standard* profile. The higher adhesive application quantities of the *New_intial_* profile can, probably, be neglected as a great deal of the adhesive had been pressed out of both *Standard* profile and *New_initial_* profile. With PUR, a higher mean f_t,0_ was achieved with the *Standard* profile. The lower mean f_t,0_ of the *New_initial_* profile can possibly be explained due to the incomplete gap filling (Figure 7) and due to the PUR’s higher ductility but lower stiffness compared to MUF and PRF [36]. In contrast, MUF and PRF filled the *New_initial_* profile and may have led to an reinforcement due to their high stiffness and gap filling properties [37,38]. The MUF and PRF contain relatively rigid, highly cross linked polymers, whereas the PUR contained more flexible polymers [36]. The stiffness of the adhesives measured by means of nanoindentation and by macroscopic testing methods presented in [39] was the highest for MUF, followed by PRF and the lowest was for PUR. The MUF can achieve a similarly high stiffness as the wood tested in this study. The mean f_t,0_ of this study were similar compared to [4] and higher compared to [5,40], which used a *Standard* profile (albeit with slightly different lamella qualities and similar adhesives). The largest standard deviations in f_t,0_ were observed in the Control and MUF*Ni*700 series. The standard deviation of MUF*Ni*700 tended toward higher f_t,0_. For MUF*Ni*700, an increase in f_t,0_ corresponded with increasing E_0_, indicating an improved ratio of the lamella strength vs. the finger-joint strength.

In the bending tests (Table 6), the MC and density (ρ) of the series were similar. All series showed a low linear relation between the bending strength (f_m_) and the ρ. The Control series reached the highest mean f_m_. The MUF*Ni*700 series achieved the highest mean global MOE (E_0,fj_). This is, again, an indicator that the stiff and gap filling MUF may have led to a reinforcement in the cross-grooved fingers of the *New_initial_* profile. The test duration of 300 (±120) seconds was met by the Control specimens and finger-jointed specimens. In most cases, it was near the lower limit for the finger-jointed lamellas.

**Figure 10:**
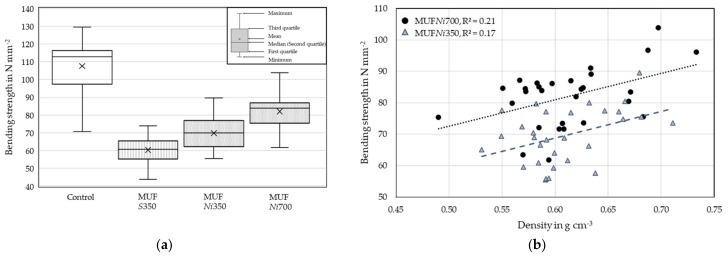
Bending strength of the finger-jointed birch and Control specimens (**a**). The relation between the density and bending strength of the MUF*Ni*700 and MUF*Ni*350 finger-jointed specimens (**b**).

Figure 10 depicts the f_m_ of the finger-jointed birch series and Control series. In addition, it shows the relation between the ρ and f_m_ of the MUF*Ni*700 and MUF*Ni*350 series.

MUF*Ni*350 achieved a 16% higher mean f_m_ and MUF*Ni*700 achieved a 36% higher mean f_m_ compared to MUF*S*350, which was finger jointed with the *Standard* profile. The adhesive application quantity of MUF*Ni*350 and MUF*S*350 was the same. This is an indicator that the *New_initial_* profile has a more even stress distribution compared to the *Standard* profile as an increase in strength was achieved even with the same application quantity of adhesive. However, a higher quantity of adhesive was required to achieve a pronounced strength increase in the *New_intial_* profile. As the *New_intial_* profile has large cross-grooved fingers with a high proportion of gaps, it was decided to shorten the length of the cross-grooved fingers for the further tests in this study. The aim was to maintain the improved stress distribution to increase the structural integrity of the profile and to reduce the quantity of adhesive that is required for complete wetting and gap filling of the profile.

In native unjointed wood, a higher density usually results in higher strengths. In [41], no correlation between the finger-joint tensile strength and the density of beech finger-jointed lamellas existed. In this study, a low relation between the finger joint f_m_ and density of MUF*Ni*700 (R^2^ = 0.21) and MUF*Ni*350 (R^2^ = 0.17) was obtained. A linear regression with R^2^ = 0.21 and 0.17 did not allow for an assessment of the dependencies. However, the two series showed an increase in f_m_ with increasing density of the specimens, and this trend was not evident for the Control and MUF*S*350 series. In the Control series, wood characteristics, e.g., grain deviations, may have overlaid the influence of the density. A larger number and equal number of specimens in the series would be necessary to further verify the validity of the correlation between the density and strength.

The tested beech lamella series (Table 7) had similar MC, density and dynamic MOE before finger jointing (E_0_). The E_0_ was at a rather low level for the beech lamellas. During the tensile test, only the span between the clamps containing fewer defects were tested. This probably led to a large difference between the relatively low E_0_ for the entire lamella, including more defects, and the relatively high tensile strength (f_t,0_), which was determined for a smaller section of the lamella. The few failures outside the finger joint were mostly due to grain deviations.

**Figure 11:**
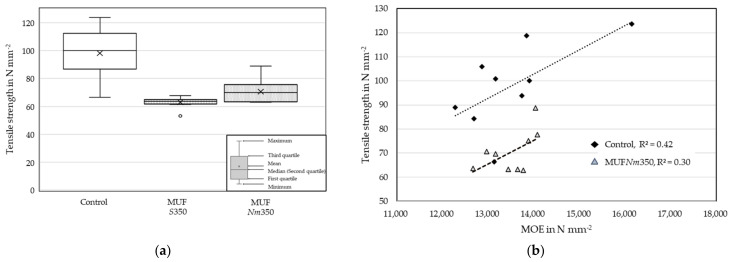
The tensile strength of the finger-jointed beech and Control lamellas (**a**). The relation between the MOE before finger jointing and the tensile strength of the Control and MUF*Nm*350 lamellas (**b**).

Figure 11 shows the f_t,0_ of the series and the linear relation between E_0_ and f_t,0_ for the MUF*Nm35*0 and the Control lamellas.

The Control series achieved the highest mean f_t,0_ with 98.1 N mm^−2^. The test duration of 300 (±120) seconds was exceeded by the Control lamellas in most cases and was often near the upper limit for the finger-jointed lamellas. The MUF*Nm*350 series achieved a mean f_t,0_ of 70.5 N mm^−2^, which was higher compared to the *Standard* profile MUF*S*350. No correlation existed between the E_0_ and f_t,0_ in the case of MUF*S*350. A low-to-moderate linear relation was found for the MUF*Nm*350 and Control series.

## 4. Conclusions

In summary, the *New* profiles tested in this study showed an increase in the tensile and bending strength compared to the *Standard* profile when the MUF and PRF adhesives were used. Curing problems were suspected with the PRF and, consequently, these results were subject to a higher degree of uncertainty. For the PUR, as for the PRF, only the *New_intial_* profile was tested. More extensive tests with different adhesives are currently not mandatory as, in this stage, the focus should be more on the geometric parameters of the *New* profile and their optimisation. Testing different adhesives increases the complexity and should be further considered and harmonised with the geometries at a later stage. The *New_modified_* profile showed advantages in manufacturing over the *New_intial_* profile. For example, when regarding the adhesive application quantity and the integrity of the finger-joint profile.

The principle of cross-grooved fingers had a positive effect on the stress distribution in the finger joint and, as a result, on the strengths. The improved stress distribution was attributed to the fewer cracks occurring in the finger joint by means of the *New* profiles. Strength graded and finger-jointed lamellas with closed finger bases are conceivable with the *New* profiles. This could be accompanied by visual advantages over the conventional structural finger joints and greater protection against the moisture penetration in the use case. According to the current state of knowledge, the *New* profiles can make a significant contribution to the usability of hardwoods as a structural building material as the ratio of the high lamella strength of the hardwoods vs. the finger-joint strength is improved. Nevertheless, the results of this study should be verified with a larger number of lamellas. Furthermore, the *New* profiles are conceivable for materials that are brittle and tend to crack. Additional analyses are needed, especially on the stress behaviour of the finger joint (e.g., finite element method, electronic speckle pattern interferometry or digital image correlation), which can be used as a basis for the further optimisation of the *New* profiles. The principle design and process for manufacturing the *New* profiles has been patented, and Weinig Grecon GmbH & Co. KG is the owner of the patent. The configuration of a finger-jointing line for the industrial production of finger joints with the *New* profiles is in the planning stage.

## Figures and Tables

**Table 1:** The density, viscosity, mixing ratio and application quantity of the adhesives.

Adhesives	Density [g cm^−3^]		Viscosity [mPa s]		Mixing Ratio (R:H)	Application Quantity ^1^ [g m^−2^]
	Resin	Hardener	Resin	Hardener		
PUR	1.16		24,000		1-comp., no primer	160
MUF	1.27	1.08	3000–10,000	1800–2800	100:20	350
PRF	1.16	1.18	5000–10,000	5000–8000	100:20	350

^1^ The recommended application quantity for conventional finger joints can be different for test series with newly developed finger-joint profiles.

**Table 2:** The series of the birch finger-jointed lamellas tested in the tensile tests.

Part 1, birch lamellas (2000 × 150 × 35 mm^3^), tensile test				
*Standard*, lj = 16.05 mm (vertical)				
*New_initial_*, lj = 16.05 mm (vertical) and 8.5 mm (horizontal)				
**Series**	**Adhesive**	**Quantity [g m^−2^]**	**Profile**	**Number of Specimens**
Control	-	-	*Unjointed*	16
PUR*S*160	PUR	160	*Standard*	16
PUR*Ni*320	PUR	320	*New_intial_*	16
MUF*S*350	MUF	350	*Standard*	16
MUF*Ni*700	MUF	700	*New_intitial_*	16
PRF*S*350	PRF	350	*Standard*	14
PRF*Ni*700	PRF	700	*New_intial_*	16

**Table 3:** The series of birch finger-jointed specimens tested in the bending tests.

Part 2, birch specimens (655 × 31 × 31 mm^3^), bending test				
*Standard*, lj = 16.05 mm (vertical)				
*New_initial_*, lj = 16.05 mm (vertical) and 8.5 mm (horizontal)				
**Series**	**Adhesive**	**Quantity [g m^−2^]**	**Profile**	**Number of Specimens**
Control	-	-	*Unjointed*	30
MUF*S*350	MUF	350	*Standard*	35
MUF*Ni*350	MUF	350	*New_initial_*	32
MUF*Ni*700	MUF	700	*New_intiial_*	32

**Table 4:** The series of beech finger-jointed lamellas tested in the tensile tests.

Part 3, beech lamellas (2000 × 100 × 25 mm^3^), tensile test				
*Standard*, lj = 16.05 mm (vertical)				
*New_modified_*, lj = 16.05 mm (vertical) and 4 mm (horizontal)				
**Series**	**Adhesive**	**Quantity [g m^−2^]**	**Profile**	**Number of Specimens**
Control	-	-	*Unjointed*	10
MUF*S*350	MUF	350	*Standard*	15
MUF*Nm*350	MUF	350	*New_modified_*	15

**Table 5:** The mean values and standard deviation (±) of the birch finger-jointed lamellas tested in the tensile tests. Replicates (N), moisture content (MC), density (ρ), dynamic MOE of lamellas before finger jointing (E_0_), dynamic MOE of finger-jointed lamellas (E_0,fj_), tensile strength (f_t_,_0_) and the coefficient of determination of linear regression (R^2^).

All Specimens						With Failure in Finger Joint		
Series	N	MC [%]	ρ [g cm^−3^]	E_0_ [N mm^−2^]	E_0,fj_ [N mm^−2^]	N	f_t,0_ [N mm^−2^]	R^2^ f_t_,_0_; E_0_
Control	16	9.1 (±0.6)	0.62 (±0.06)	14,550 (±1785)	-	16	79.9 (±15.1)	0.56
PUR*S*160	16	9.1 (±0.4)	0.61 (±0.05)	14,550 (±1758)	15,061 (±1840)	16	61.2 (±6.6)	0.06
PUR*Ni*320	16	9.1 (±0.5)	0.61 (±0.04)	14,550 (±1658)	15,114 (±1705)	15	52.8 (±7.3)	0.16
MUF*S*350	16	9.2 (±0.5)	0.61 (±0.05)	14,549 (±1648)	15,112 (±1752)	12	57.0 (±5.5)	0.18
MUF*Ni*700	16	9.1 (±0.5)	0.63 (±0.04)	14,550 (±1667)	15,079 (±1774)	14	67.2 (±14.7)	0.72
PRF*S*350	14	9.0 (±1.3)	0.61 (±0.06)	14,236 (±1334)	-	13	36.8 (±7.3)	0.01
PRF*Ni*700	16	8.9 (±1.1)	0.59 (±0.03)	14,368 (±1554)	-	15	44.0 (±9.0)	0.30

**Table 6:** The mean values and standard deviation (±) of the birch finger-jointed specimens tested in the bending tests. Replicates (N), moisture content (MC), density (ρ), global MOE of the finger-jointed specimens (E_0,fj_), bending strength (f_m_) and the coefficient of determination of linear regression (R^2^).

Series	N	MC [%]	ρ [g cm^−3^]	E_0,fj_ [N mm^−2^]	f_m_ [N mm^−2^]	R^2^ f_m_; p
Control	30	7.3 (±0.18)	0.63 (±0.06)	10,006 (±1535)	107.5 (±14.2)	0.10
MUF*S*350	35	7.3 (±0.18)	0.62 (±0.05)	9524 (±1204)	60.4 (±6.8)	0.06
MUF*Ni*350	32	7.5 (±0.22)	0.61 (±0.04)	9845 (±1210)	69.9 (±8.5)	0.17
MUF*Ni*700	32	7.1 (±0.20)	0.61 (±0.05)	10,070 (±1267)	82.0 (±9.0)	0.21

**Table 7:** The mean values and standard deviation (±) of the beech finger-jointed lamellas tested in the tensile tests. Replicates (N), moisture content (MC), density (ρ), the dynamic MOE of lamellas before finger jointing (E_0_), tensile strength (f_t_,_0_) and the coefficient of determination of linear regression (R^2^).

All Specimens					With Failure in Finger Joint		
Series	N	MC [%]	ρ [g cm^−3^]	E_0_ [N mm^−2^]	N	f_t,0_ [N mm^−2^]	R^2^ f_t_,_0_; E_0_
Control	10	11.7 (±0.45)	0.69 (±0.02)	13,188 (±1436)	9	98.1 (±16.5)	0.42
MUF*S*350	15	11.9 (±0.27)	0.69 (±0.01)	13,267 (±614)	11	63.1 (±3.6)	0.01
MUF*Nm*350	15	11.9 (±0.29)	0.69 (±0.02)	13,326 (±547)	10	70.5 (±7.9)	0.30

## Data Availability

Data are contained within the article.
